# Narcolepsy in early childhood: a case report and a Mini review

**DOI:** 10.3389/fped.2024.1475029

**Published:** 2025-01-07

**Authors:** Guorui Liu, Guanxiong Li, Yihao Wang, Ming Yin, Wen Pan, Yu Zhao, Shigeng Gao, Peiqi Shi, Jing Wen, Xiao Pan, Yajing Wang, Yanfei Zhang

**Affiliations:** ^1^ Department of Medical Psychology, No. 905 Hospital of PLY Navy, Shanghai, China; ^2^ Sleep Medicine Center, Suzhou Guangji Hospital, Suzhou, China; ^3^ Department of Medical Psychology, Second Affiliated Hospital of Naval Medical University, Shanghai, China

**Keywords:** children, narcolepsy, treatment, cataplexy, case report

## Abstract

Narcolepsy is a sleep-wake disorder with an onset commonly seen in individuals aged 10–30 years. Due to various reasons, the diagnosis of narcolepsy often experiences a delay of at least ten years. Diagnosing narcolepsy in children is particularly challenging due to atypical symptoms, leading to frequent misdiagnosis or missed diagnoses. We report a case of narcolepsy in a four-year-old girl to provide insights into the early diagnosis and treatment of narcolepsy in children. As a chronic condition, narcolepsy can lead to decreased quality of life, including psychological issues such as depression and anxiety. Furthermore, there are few randomized controlled trials involving pediatric narcolepsy patients. To provide a comprehensive treatment approach for pediatric narcolepsy, we review the current progress in the treatment of narcolepsy in children.

## Introduction

Narcolepsy is a sleep-wake disorder characterized by irresistible daytime sleepiness, cataplexy, sleep paralysis, and hypnagogic or hypnopompic hallucinations as primary clinical features (1). However, these symptoms do not appear simultaneously in most cases, complicating the diagnosis. The onset of narcolepsy is commonly seen in individuals aged 10–30 years and can lead to social function impairments, including in learning, work, life, and social interactions (2, 3). Early diagnosis allows timely intervention, helping patients improve their quality of life.

Compared to the well-established diagnostic criteria of modern medicine, the diagnostic standards for narcolepsy have only been established relatively recently (4). There have been reports of narcolepsy cases as early as infancy, but various factors contribute to the frequent misdiagnosis or missed diagnosis in preschool children. Retrospective studies indicate that narcolepsy patients exhibit related clinical symptoms in infancy, and research shows a diagnostic delay of at least ten years for narcolepsy (3, 5). Therefore, we report a case of narcolepsy in a four-year-old girl to provide insights into the early diagnosis and treatment of narcolepsy in children and review the literature on the treatment of pediatric narcolepsy.

## Case report

A four-year-old girl presented with excessive daytime sleepiness over the past few months. Recently, she often felt sleepy during the day and would fall asleep uncontrollably. She frequently mentioned feeling sleepy halfway through meals and would immediately fall asleep for a few minutes to over ten minutes, waking up refreshed. Occasionally, she would drop her utensils while eating, her head would drop, and she would quickly open her eyes when called. Sometimes, there were instances of unsteadiness when laughing, drooping eyelids, difficulty speaking, and an inability to lift the head (due to decreased neck muscle tone), sometimes accompanied by tongue protrusion. In class, there were episodes of being unable to keep the eyes open, drowsiness, and easy fatigue.

The condition worsened, with an increased frequency of uncontrollable daytime sleep episodes. She also began talking in her sleep at night, sleeping 9–10 h each night. Her kindergarten teacher reported that she often dozed off in the morning and needed to nap about three times in the afternoon after returning home, after which her energy improved. Weeks later, she continued to feel sleepy upon waking and became irritable and prone to anger. Neurological examinations showed no abnormalities, and a head MRI revealed no positive findings.

**Figure 1:**
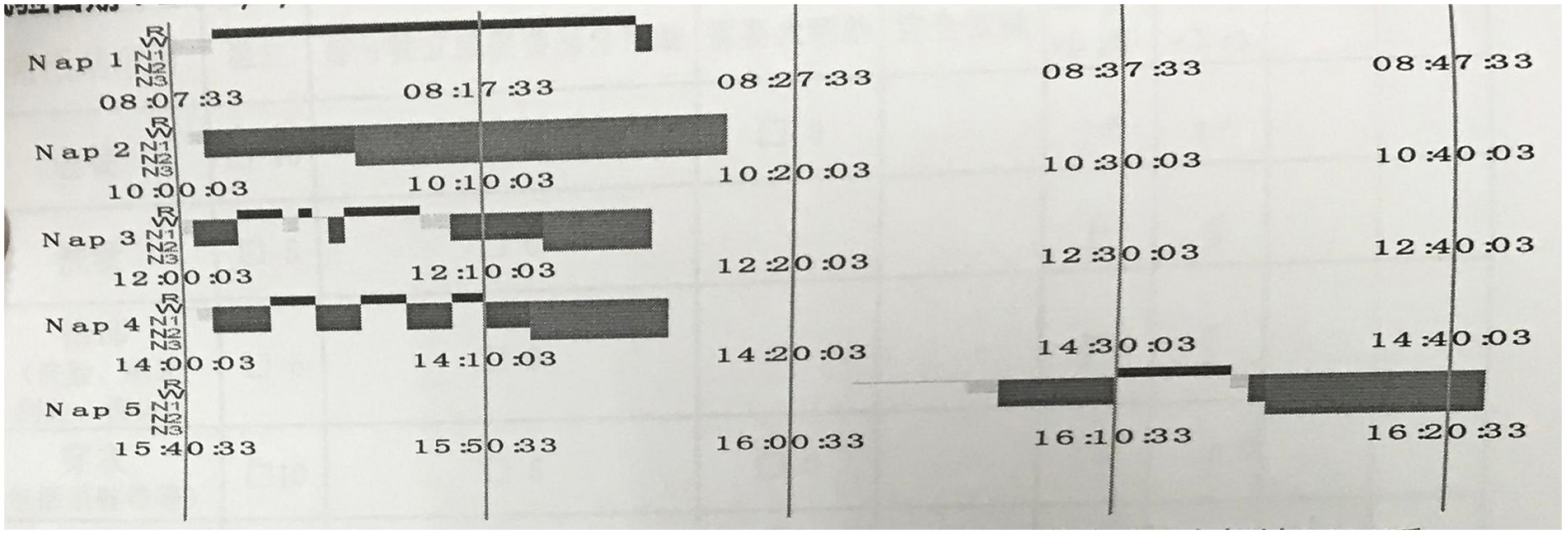
PSG results showed: REM latency is 5.5 min.

**Figure 2:**
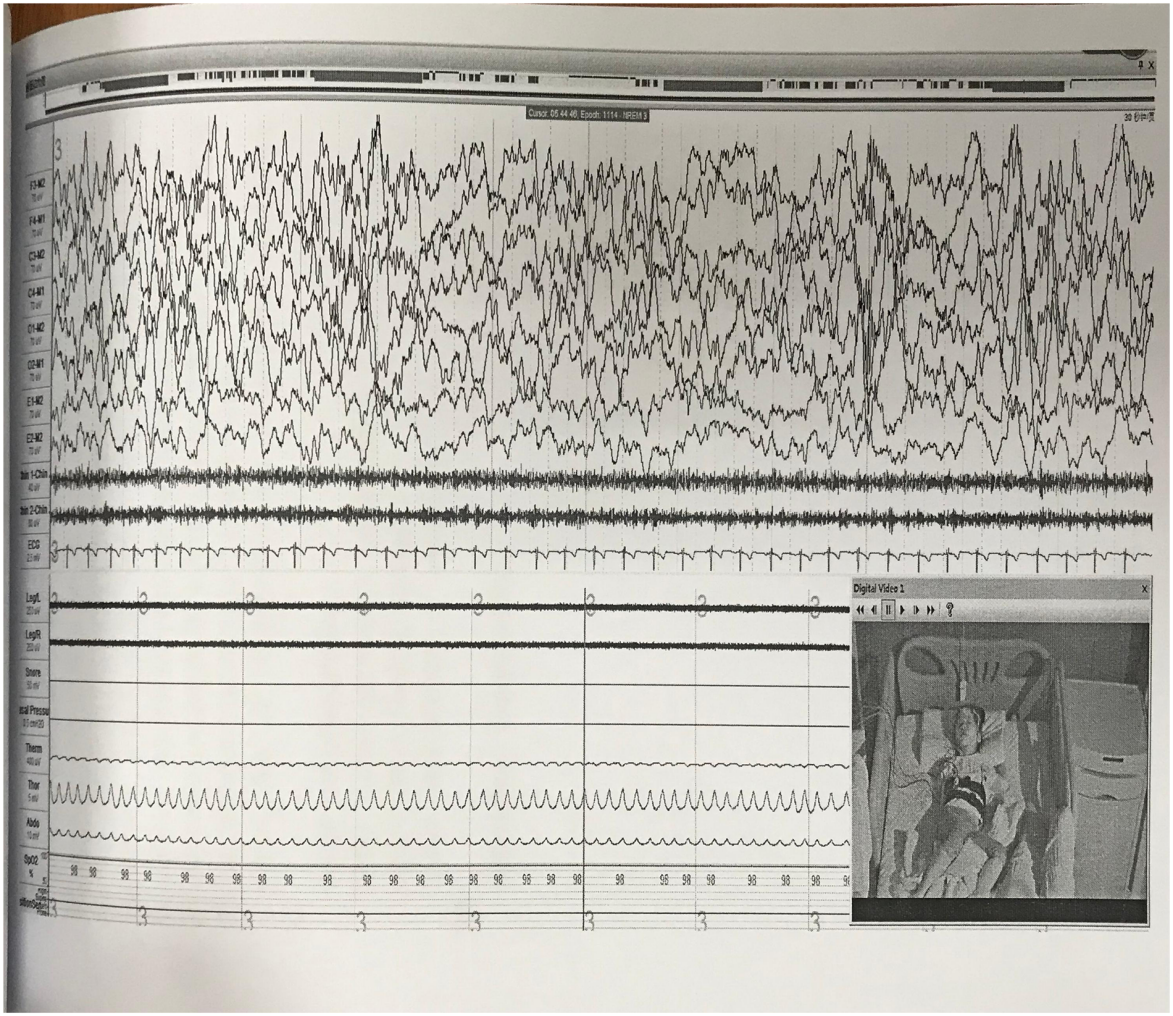
The MSLT showed a mean sleep latency of 5.3 min and a mean REM sleep latency of 2.6 min; four sleep-onset REM episodes were observed.

A 24-hour ambulatory electroencephalogram (EEG) showed no epileptiform discharges or other abnormal brain electrical activity. An overnight polysomnography (PSG) with video (Figure 1, Table 1) revealed a sleep latency of 0.5 min and a REM latency of 5.5 min. A Multiple Sleep Latency Test (MSLT) reported five sleep episodes during five monitoring sessions, with an average sleep latency of 5.3 min and four instances of sleep-onset REM periods (Figure 2).

**Table 1:** Summary of nocturnal polysomnography.

Total recording time	601.0 min
Sleep period	536.0 min
Wake after sleep onset	57.0 min
Total sleep time	536.0 min
Sleep onset	57.5 min
Sleep efficiency	89.20%
Number of awakenings	46
Non-rapid eye movement 1	62.0 min
Non-rapid eye movement 2	146.0 min
Non-rapid eye movement 3	196.0 min
Rapid eye movement	132.0 min
Apnea + Hypopnea	0.2
Obstructive apnea	0
Central apnea	0.2
Mixed apnea	0
Limb movement	0

Based on the neurological examination and imaging results, we excluded epilepsy (absence seizures). According to the patient's symptoms, PSG, and MSLT results, the diagnosis is narcolepsy with cataplexy. The family and the girl were provided with education on sleep hygiene, including maintaining a regular sleep schedule and planning short naps. The patient was prescribed venlafaxine extended-release capsules at a dose of 18.75 mg once daily. A follow-up after one month indicated significant improvement in daytime sleepiness and cataplexy symptoms. Continued medication led to another follow-up after three months, which showed sustained positive effects.

## Discussion

The typical clinical manifestations of narcolepsy include irresistible daytime sleepiness, cataplexy, sleep paralysis, and hypnagogic or hypnopompic hallucinations (6). The etiology of narcolepsy remains unclear, though current research suggests it may be related to genetic, environmental, and infectious factors. Studies on brain structure have indicated that it might be associated with decreased function of the ascending reticular activating system in the brainstem or hyperactivity of the caudal pontine reticular nucleus (7, 8). Based on the presence or absence of cataplexy, narcolepsy is classified into narcolepsy with cataplexy (narcolepsy type 1, NT1) and narcolepsy without cataplexy (narcolepsy type 2, NT2). The clinical manifestation of cataplexy involves a sudden, temporary loss of bilateral muscle tone triggered by strong emotions, while maintaining clear consciousness. Studies have shown that 90% of patients with NT1 are HLA-DQB1*06:02 positive (9), while only 10%–20% of non-cataplexy patients exhibit this marker. Further research found that HLA-DQB1*03:01 is associated with the onset of NT1 as early as age 3, and recent NT1 cases in children have shown an increased positivity rate for both DQB1*03:01 and DQB1*06:02 (10). Additionally, in individuals who are HLA-DQB1*06:02 positive, HLA-DPB1*04:02 is associated with a reduced risk of NT1, while DPB1*05:01 increases the risk.

Narcolepsy is often accompanied by other issues. For instance, NT1 is frequently associated with comorbid eating disorder symptoms, primarily in the form of night eating syndrome (11). Compared to normal controls, NT1 patients have a higher risk of depression, anxiety symptoms (12), and suicidal thoughts. Studies on children with NT1 have found the presence of psychiatric issues, such as attention-deficit/hyperactivity disorder (ADHD), mood disorders (depression and anxiety), and learning difficulties, likely due to excessive daytime sleepiness, persistent and distressing sleep-related hallucinations, and the psychological stress of the disease burden (13). These issues highlight the need for targeted interventions for narcolepsy.

As a lifelong sleep disorder, the peak onset ages for narcolepsy are 15 and 35 years. However, previous studies have reported numerous cases in children under 10, indicating that this condition is not uncommon among pre-adolescent children, and it can also occur in preschool-aged children. Cases have even been reported in children under the age of 2 (9).

Various factors contribute to the frequent misdiagnosis or missed diagnosis of narcolepsy in preschool children, summarized as follows:

**Complex and Atypical Symptoms:** Clinically, only 10%–15% of patients exhibit all four major features of narcolepsy, and this is even rarer in children (5). Increased daytime sleepiness, a prominent symptom in pediatric narcolepsy, is often mistaken for normal daytime napping or attributed to laziness and inattentiveness, delaying the diagnosis (3). Daytime sleep attacks, where the patient can be awakened or seems hysterical, are often misdiagnosed as hysterical episodes. Atypical cataplexy, including cataplectic-like events and cataplectic facies, is another issue (14). Cataplectic-like events are sudden episodes of muscle weakness not related to emotional triggers, occurring frequently, sometimes dozens of times a day. When asked about sleepiness, patients often deny it, leading to misdiagnosis as epilepsy (atonic seizures) or other muscle weakness disorders (e.g., myasthenia gravis, peripheral neuropathy, myopathy). Cataplectic facies, which involve facial muscle involvement like drooping eyelids, mouth opening, tongue protrusion, and facial relaxation, often appear early in the disease course and can be mistaken for epilepsy (partial status epilepticus) or tics (5).
**Associated Symptoms:** Narcolepsy can also manifest as increased appetite, obesity, mood disorders, aggression, or anxiety, often mistaken for laziness, depression, or even epilepsy (3). The atypical symptoms often lead parents to repeatedly visit various hospital departments, where clinicians might not consider narcolepsy, resulting in missed or incorrect diagnoses (5). Additionally, the young age of onset and the inability of children to accurately describe their symptoms contribute to misdiagnosis.

The current diagnostic criteria for narcolepsy are a mean sleep latency of ≤8 min on the Multiple Sleep Latency Test (MSLT) and the presence of ≥2 Sleep Onset Rapid Eye Movement Periods (SOREMPs). However, due to the lack of highly specific biomarkers, relying solely on MSLT may result in “false positives” (15, 16). Since our hospital has not yet implemented testing for thalamic secretin and HLA typing, one limitation in this case is the absence of certain tests, including HLA. Future research can focus on conducting more comprehensive testing for related cases.

## Pharmacological treatment of pediatric narcolepsy

The pharmacological treatment of pediatric narcolepsy primarily focuses on managing daytime sleepiness and cataplexy. Few randomized controlled trials involve pediatric narcolepsy patients. Among all medications, only sodium oxybate is approved by the FDA and EMA for treating daytime sleepiness and cataplexy in children and adolescents (17).

### Modafinil

Studies have shown that modafinil can alleviate daytime sleepiness, with higher doses being more effective than lower ones. Split dosing may provide more sustained effects than a single dose (18). Research on pediatric narcolepsy indicates that the dosage range for children is similar to that for adults, ranging from 200 to 600 mg/day, usually administered in split doses to alleviate afternoon and evening drowsiness. Children taking modafinil did not report side effects such as headaches, nausea, or sweating in relevant cases (18–20).

### Methylphenidate

Methylphenidate alleviates daytime sleepiness and is available in short-acting and long-acting formulations. The starting dose is 5 mg/day, slowly titrated to a maximum of 60 mg/day, with no single dose exceeding 25 mg (4).

### Amphetamines

The starting dose for amphetamines is 2.5 mg/day, with a slow titration of 2.5–5 mg/week, not exceeding a maximum of 40 mg/day.

### Sodium oxybate

Sodium oxybate is the only medication approved by the FDA and EMA for treating daytime sleepiness and cataplexy in children and adolescents aged 7 and older (4, 5). Common side effects include nausea, dizziness, sleepwalking, and headaches (21). The initial dose is 2.25 g at bedtime, followed by another 2.25 g 2.5–4 h later, gradually increasing by 1.5 g/week to an effective dose of 6–9 g per night.

### Pitolisant

Limited studies have reported that pitolisant can alleviate daytime sleepiness in children and adolescents, with a typical dose range of 4.5–36 mg/day. For children with low body weight, the dose can be halved. Side effects include insomnia, headaches, and hallucinations, with most, except insomnia, gradually subsiding (17, 21, 22).

### Tricyclic antidepressants

Clomipramine is often used to alleviate cataplexy symptoms, with common side effects including dry mouth, drowsiness, orthostatic hypotension, and weight gain. Sudden discontinuation can worsen symptoms. The starting dose is 25 mg/day, adjusted weekly to a final dose of 3 mg/kg/day or 200 mg/day (17, 23). However, some literature suggests that clomipramine has limited efficacy for cataplexy (4). Other tricyclic antidepressants include imipramine and protriptyline.

### Selective serotonin reuptake inhibitors (SSRIs)

Fluoxetine is commonly used to alleviate cataplexy symptoms, with side effects such as headaches, anxiety, nervousness, and personality changes. The starting dose is 10 mg/day, with a maximum dose of 20 mg/day (4). Other SSRIs include fluvoxamine.

### Serotonin and norepinephrine reuptake inhibitors (SNRIs)

Data on venlafaxine use in children are limited, with only two-year follow-up case studies available. It can alleviate cataplexy symptoms in narcolepsy, with side effects similar to those of SSRIs. The starting dose is 37.5 mg/day, with a maintenance dose of 75–300 mg/day (17). Clinicians often prefer SSRIs and SNRIs, but abrupt discontinuation can lead to rebound cataplexy (5).

## Other treatments

### Immunotherapy

Some studies suggest a connection between NT1 and autoimmune mechanisms, with intravenous immunoglobulin G (IVIG) potentially alleviating symptoms. However, research is limited and focused on early-stage treatment, with results remaining controversial. Further data is needed to exclude the possibility of spontaneous symptom remission in narcolepsy (6, 17, 23).

### Non-pharmacological treatments

Children with narcolepsy often face numerous psychological issues, such as declining academic performance, reduced motivation, school aversion, and anxiety (24–26). Non-pharmacological treatments, as adjunctive or alternative therapies, are frequently used to alleviate daytime sleepiness. Some studies report that medication alone may not fully relieve daytime sleepiness, with only about 15% of narcolepsy patients relying solely on medication (27, 28). Over half of the patients require adjunctive non-pharmacological treatments (26). These include cognitive-behavioral therapy (CBT) and behavioral therapy, with clinical guidelines from various sleep medicine or neurology associations including recommendations such as regular napping, sleep hygiene, a balanced diet, and physical activity (29–32).

### Behavioral management

Studies suggest that scheduled naps at specific times during the day can alleviate daytime sleepiness (two to three short naps of 15–20 min each). Nap duration should be controlled to avoid sleep inertia from prolonged naps (33, 34). However, some studies found no significant difference between short and long naps in aspects such as reaction speed after waking (35).

Additionally, extending nighttime sleep can reduce daytime sleep pressure, alleviating daytime sleepiness. Physical activity can also reduce weight and alleviate daytime sleepiness without triggering cataplexy (36, 37). Other common behavioral strategies to alleviate daytime sleepiness include environmental management (e.g., keeping the environment dry and cool, enhancing ventilation), dietary management (e.g., avoiding overeating, consuming vitamin-rich foods), staying active (e.g., participating in activities, avoiding prolonged sitting), and using pain to stay awake (e.g., pinching oneself) (26, 28).

### Cognitive-behavioral therapy (CBT)

CBT has been more commonly applied to chronic insomnia, known as Cognitive-Behavioral Therapy for Insomnia (CBT-I). It has further developed into various forms, including Internet-based CBT for Insomnia (ICBT-I). Despite behavioral management being recommended for pediatric narcolepsy, research on the effectiveness of CBT for narcolepsy is limited (17). Researchers have developed a novel CBT for narcolepsy, involving online assessments and remote interventions through different modules (24).

CBT can be used as a non-pharmacological treatment to alleviate cataplexy, daytime sleepiness, and sleep-related hallucinations. The treatment mainly comprises three parts: (1) improving sleep disturbances through behavioral management; (2) altering beliefs, motivations, and emotions that play a significant role in maintaining narcolepsy through cognitive therapy; (3) educating patients about the disease, including its etiology, symptoms, and medication precautions. Specific methods include systematic desensitization, stimulus control, and imagery rehearsal therapy (37).

### Psychological counseling and support groups

Some patients with narcolepsy also experience anxiety, depression, and other emotional issues, which can be alleviated through psychological counseling. Studies have shown that NT1 patients tend to use fewer active coping strategies when dealing with the symptoms of narcolepsy. Instead, they are more likely to exhibit mental and behavioral disconnection from real life, such as mentally distancing themselves from stressors or using passive and avoidant approaches to handle problems rather than actively attempting to resolve them (38). Research has also shown that when chronic illness leads to a decline in quality of life, peer support can help patients combat feelings of isolation, gain psychosocial support, boost their confidence in overcoming the illness, and alleviate negative emotions (1, 3, 39). Certain associations for narcolepsy, established either officially or privately, assist patients and their families by providing professional knowledge about the diagnosis and treatment of the disease and opportunities for narcolepsy patients to connect with each other (17, 40). Therefore, for the treatment of narcolepsy, relying solely on medication is insufficient; it requires supplemental behavioral and psychological therapies in addition to medication.

In summary, children suspected of having narcolepsy should be referred to a multidisciplinary sleep medicine center. For diagnosed pediatric narcolepsy patients, different treatment plans should be adopted based on their symptoms. For those without cataplexy, medication combined with behavioral management is recommended. For patients with cataplexy, or those whose quality of life is compromised, or who have anxiety and depression, medication combined with CBT is suggested. Currently, sodium oxybate is the only medication approved by the FDA for alleviating cataplexy. However, administering sodium oxybate before the patient goes to bed may pose a risk of nighttime falls. Additionally, a second dose is required 3–4 h after sleep onset, and there is a potential risk of side effects such as sleepwalking and enuresis. Other medications, including venlafaxine, atomoxetine, and fluoxetine, have been used in clinical practice, with careful monitoring of relevant test results being essential. Studies have shown that venlafaxine is highly effective in alleviating cataplexy and has been recommended as a first-line treatment for adult cataplexy. Furthermore, researchers have used venlafaxine in children and adolescents aged 7–14, finding it effective in relieving cataplexy without delayed effects (41, 42). Based on these findings, we opted for low-dose venlafaxine in this case. Future studies with larger sample sizes and control groups are warranted. Additionally, forming peer support groups can help patients and their families understand the disease better. Disease associations can provide education on medications and enhance patients' and families' medication adherence, ensuring safe pharmacological treatment (43).

## Data Availability

The datasets presented in this article are not readily available because of ethical and privacy restrictions. Requests to access the datasets should be directed to the corresponding authors.
